# Rustrela Virus as Putative Cause of Nonsuppurative Meningoencephalitis in Lions

**DOI:** 10.3201/eid2905.230172

**Published:** 2023-05

**Authors:** Madeleine de le Roi, Christina Puff, Peter Wohlsein, Florian Pfaff, Martin Beer, Wolfgang Baumgärtner, Dennis Rubbenstroth

**Affiliations:** University of Veterinary Medicine Hannover Foundation, Hannover, Germany (M. de le Roi, C. Puff, P. Wohlsein, W. Baumgärtner);; Friedrich-Loeffler-Institut, Greifswald-Insel Riems, Germany (F. Pfaff, M. Beer, D. Rubbenstroth)

**Keywords:** rustrela virus, Rubivirus, *Rubivirus strelense*, lions, viruses, meningoencephalitis, retrospective study, meningitis/encephalitis, Germany, *Suggested citation for this article*: de le Roi M, Puff C, Wohlsein P, Pfaff F, Beer M, Baumgärtner W, et al. Rustrela virus as putative cause of nonsuppurative meningoencephalitis in lions. Emerg Infect Dis. 2023 May [*date cited*]. https://doi.org/10.3201/eid2905.230172

## Abstract

Retrospective investigation of archived tissue samples from 3 lions displaying nonsuppurative meningoencephalitis and vasculitis led to the detection of rustrela virus (RusV). We confirmed RusV antigen and RNA in cortical neurons, axons, astrocytes and Purkinje cells by reverse transcription quantitative PCR, immunohistochemistry, and in situ hybridization.

Until recently, rubella virus (RuV), an RNA virus with single-stranded RNA genome of positive orientation, was considered the only virus of the genus *Rubivirus* and the family *Matonaviridae* (*1*–*3*). Two close relatives of RuV, designated rustrela virus (RusV) and ruhugu virus (RuhV), were discovered in animals in 2020. RusV was demonstrated to cause nonsuppurative meningoencephalitis in a range of zoo animals in Germany (*1*,*4*,*5*). Furthermore, RusV was detected in domestic cats (*Felis catus*) showing clinical signs of staggering disease in Germany, Austria, and Sweden (*6*). Wild yellow-necked field mice (*Apodemus flavicollis*) and wood mice (*Apodemus sylvaticus*) are assumed to be reservoir hosts of RusV (*1*,*4*,*6*).

In the 1970s and 1980s, a series of fatal nonsuppurative encephalitis cases of undetermined cause had occurred in lions (*Panthera leo*) and tigers (*Panthera tigris*) kept in zoological gardens in Germany (*7*,*8*). We reinvestigated cases of 3 lions with nonsuppurative meningoencephalitis of unknown etiology from the 1980s for the presence of RusV.

The etiology of meningoencephalitides remains undetermined in many cases (*6*,*9*); a possible explanation is that conventional methods based on the recognition of virus-specific proteins and nucleic acids did not detect viral variants. Immunohistochemical detection of double-stranded RNA (dsRNA) is considered a virus-sensing tool irrespective of the particular virus (*10*). Therefore, we assessed the applicability of antibodies sensing dsRNA as an alternative virus detection method.

## The Study

**Figure 1:**
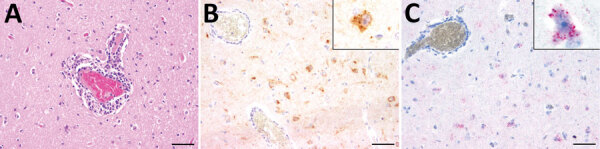
Histopathologic, immunohistochemical, and in situ hybridization findings in the cerebrum of a lion tested positive for rustrela virus (RusV) by quantitative reverse transcription PCR. A) Histopathologic analysis of cerebral sample from lion 3 indicated lymphohistiocytic meningoencephalitis and vasculitis. B) Immunohistochemistry analysis showed RusV antigen in cortical neurons and their processes. Cytoplasmic granular immunoreactivity is visible (inset). C) In situ hybridization revealed RusV RNA in cortical neurons; we observed cytoplasmic granular-positive signal (inset). Scale bars indicate 50 µm.

We retrospectively investigated 3 lions for the presence of RusV. The lions were identified in 2 zoos in northern and western Germany; they exhibited neurologic signs and nonsuppurative meningoencephalitis. Lion 1 died in 1980 in a zoo in Lower Saxony, whereas lions 2 and 3 were submitted for pathological examination in 1989 by a zoo in North Rhine-Westphalia. All 3 lions displayed a mild, multifocal, lymphohistiocytic meningoencephalitis and vasculitis (Figure 1, panel A) and occasional glial nodules. Inflammatory infiltrates were most prominent in the cerebral gray matter and less prominent in cerebral white matter, cerebellum, and meninges. The spinal cord was not available for analysis. We tested archived formalin-fixed, paraffin-embedded (FFPE) tissues for the presence of RusV RNA and antigen by quantitative reverse transcription PCR (qRT-PCR), in situ hybridization (ISH) and immunohistochemistry (IHC). We included FFPE tissues originating from 8 lions without nonsuppurative meningoencephalitis (lions 4–11) as controls (Appendix).

FFPE brain samples from lions 1–3 tested positive for RusV RNA by the broadly reactive qRT-PCR assay panRusV-2 (*6*). Cycle quantification (Cq) values were 29–38. We detected no RusV RNA in central nervous system (CNS) samples from any of the 8 control animals (Appendix Table 1).

**Figure 2:**
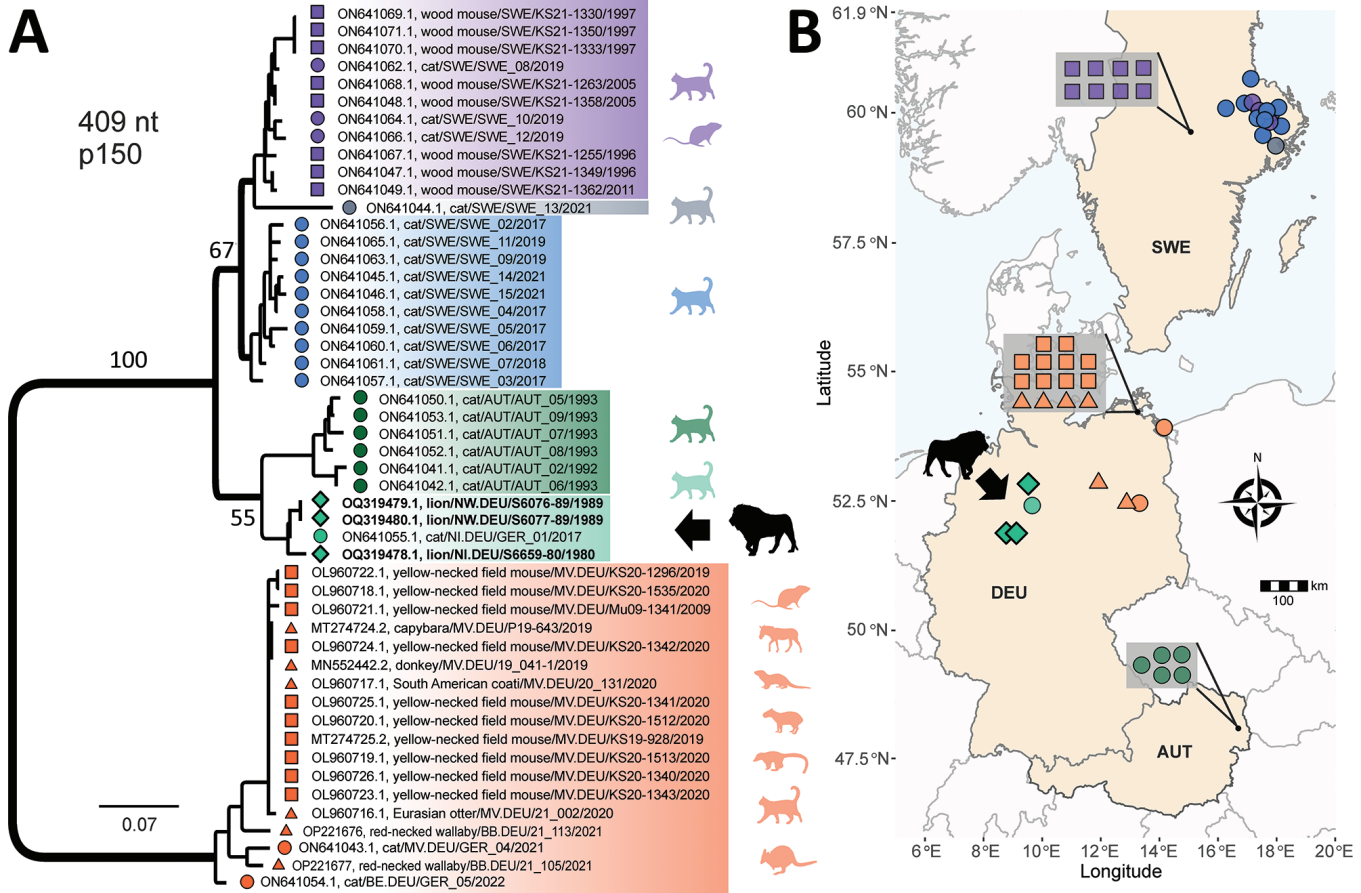
Phylogenetic analysis and spatial distribution of rustrela virus infections in Europe. A) Maximum-likelihood phylogenetic tree of partial rustrela virus (RusV) sequences (409 nt, representing genome positions 100–508 of donkey-derived RusV reference genome, GenBank accession no. MN552442.2). Only bootstrap values at major branches are shown in the phylogenetic tree. RusV sequence names are shown in the format host/ISO 1366 code of location (federal, state, country)/animal ID/year. The tree was produced using IQ-TREE version 2.2.0; transition model 2 plus empirical frequency plus gamma 4 with 1,000 bootstrap replicates. Bold text indicates sequences from this study. Scale bar indicates substitutions per site. B) Mapping of the geographic origin of RusV-positive animals in Europe. Colors represent the phylogenetic clades of the sequences. Diamonds represent lions; circles, domestic cats; triangles, other zoo animals; squares, *Apodemus* spp. rodents. Symbols in gray boxes represent individuals from the same or very close locations. AUT, Austria; BE, Berlin; DEU/GER, Germany; MV, Mecklenburg–Western Pomerania; NI, Lower Saxony; NW, North Rhine–Westphalia; SWE, Sweden.

We determined a partial host-genome RusV sequence 409 bp long for all 3 RusV-positive animals by Sanger sequencing of overlapping RT-PCR products (Appendix). The sequences shared 97.8% nucleotide identity; phylogenetic analysis revealed all 3 sequences to form a single clade together with the sequence from a domestic cat in Hannover, Lower Saxony, in 2017 (*6*). Of note, this subclade was more closely related to sequences from cats with staggering disease in Austria than to sequences from zoo animals, domestic cats, and wild rodents in northeastern Germany (Figure 2).

IHC investigation for the presence of RusV capsid antigen using monoclonal antibody 2H11B1 (*6*) revealed multifocal, cytoplasmic, granular reactions, predominantly in cerebral cortical perikarya and their axons, in few astrocytes as well as in Purkinje cells of all 3 PCR-positive lions (Figure 1, panel B). Likewise, we detected RusV-specific RNA using a newly designed ISH probe (Appendix) in the brains of lions 2 and 3, but not of lion 1. We found viral RNA as a cytoplasmic granular signal in cortical perikarya (Figure 1, panel C). We observed RusV-specific capsid antigen and RNA in cerebral cortical neurons adjacent to perivascular infiltrates and also in neurons in more distant areas not associated with inflammatory changes. Neither IHC nor ISH revealed positive signals in any of the examined peripheral organs of the 3 RusV-positive animals and RusV-negative lion 7 (Appendix Table 1) or in the CNS of control animals. IHC staining for dsRNA using the dsRNA antibodies K1 and J2 (*11*) provided positive results in the CNS of all tested animals (Appendix Table 1). Immunolabeling with anti-dsRNA antibody 9D5 (*11*) remained negative for all 3 RusV-positive animals, whereas the RusV-negative lions 7 and 9 tested positive (Appendix Table 1).

## Conclusions

The results of this study strongly indicate RusV as the potential cause of fatal lymphohistiocytic meningoencephalitis in lions from Germany in the 1980s. The animals were reported to have had neurologic disorders characterized by fever, depression, ataxia, and prolapse of the tongue (*7*). These clinical and histopathological findings are similar to those described previously for RusV-infected zoo animals and domestic cats (*1*,*4*–*6*); they also resemble RuV-induced encephalitis in humans (*12*).

A partial colocalization of RusV antigen and RNA detection with histopathologic lesions has been observed previously (*1*,*4*,*6*). Although the pathogenesis of RusV infection has not been elucidated, a virally triggered immune response that remains present even after focal virus clearance may provide an explanation for this phenomenon (*13*). In addition, vasculitis caused by a type III hypersensitivity reaction should be considered.

The lack of viral antigen and RNA in organs other than the CNS of the infected lions is consistent with previous findings in RusV-infected zoo animals of other species, in which RusV RNA was predominantly detected in the CNS and only sporadically in other organs (*1*,*4*,*5*). These results indicate a strong neurotropism of RusV also in lions.

In this study, we consistently detected RusV RNA and antigen in the affected animals using 3 independent methods (qRT-PCR, ISH, and IHC). The lack of viral RNA detection by ISH in the brain of lion 1, positive by qRT-PCR, could be a result of lower sensitivity of ISH (*14*) or of a higher degree of RNA degradation in this >40-year-old sample. Furthermore, the crosslinking of proteins caused by formalin has been shown to influence the quality and accessibility of DNA or RNA in FFPE material (*15*).

Immunohistochemical investigation for dsRNA revealed positive results in the brains of all investigated lions, regardless of their RusV infection status. Although it is possible that the RusV-negative lions were infected by other neurotropic RNA viruses, this scenario appears unlikely because no CNS lesions were observed in control animals 4–11. Thus, this method appears unsuitable for reliable detection of viral dsRNA in the brains of lions and perhaps other animals.

In summary, our study reveals that RusV was present in northern and western Germany in the 1980s. Detecting RusV in lions indicates an even broader host range of RusV, encompassing a variety of different species (*1*,*4*–*6*) and suggests that other wild and captive felids may be susceptible to RusV infection. As described previously (*6*), fulfilling Henle-Koch’s postulates by experimental reproduction of the disease has not been possible because of lack of RusV isolates. Nevertheless, the association between RusV detection and disease demonstrated in this study, combined with previous studies on RusV infections in zoo animals and domestic cats, strongly suggests RusV as a causative agent of meningoencephalitis in lions.

Appendix Additional information on rustrela virus as putative cause of nonsuppurative meningoencephalitis in lions.
