# Supplementary material for: Rustrela Virus as Putative Cause of Nonsuppurative Meningoencephalitis in Lions
Source: Emerg Infect Dis. 2023 May;29(5):1042–5. doi: 10.3201/eid2905.230172 (PMC10124629; doi:10.3201/eid2905.230172)
Supplement: Appendix — Additional information on rustrela virus as putative cause of nonsuppurative meningoencephalitis in lions. [file 23-0172-Techapp-s1.pdf]

Article DOI: https://doi.org/10.3201/eid2905.230172

Rustrela Virus as Putative Cause of Nonsuppurative Meningoencephalitis in Lions

Appendix

Materials and Methods

Animals

Archived formalin-fixed, paraffin-embedded (FFPE) material originated from 11 lions (*Panthera leo*) (Appendix Table 1). All 11 animals had been submitted from 1980 to 2022 by zoological institutions from northern and western Germany (federal states Lower Saxony and North Rhine-Westphalia) for pathological examination. Lions 1 to 3 had been selected due to the diagnosis of nonsuppurative meningoencephalitis. Lions 2 and 3 had been previously described by Truyen et al. (*1*). Lions 4 to 11 did not show signs of a nonsuppurative meningoencephalitis and were included as control animals (Appendix Table 1).

Histopathology

This study focused on the examination of FFPE tissue samples from the central nervous system (CNS). Organ samples were collected during necropsy and fixed in 10% neutral buffered formalin (NBF) for at least 24 hours. For histopathological examination, samples were processed routinely and sections were stained with Hematoxylin and eosin (HE).

RNA Extraction and RT-qPCR

Total RNA from FFPE brain sections was extracted using the miRNeasy FFPE kit (Qiagen, Hilden, Germany) following the manufacturer's instructions. During the RNA extraction, the preparations were spiked with a defined copy number of in vitro-transcribed RNA of the eGFP gene to serve as an extraction and amplification control during reverse transcription quantitative PCR (RT-qPCR), as described previously (*2*).

Rustrela virus (RusV) RNA was detected by the specific RT-qPCR assay panRusV-2 targeting the consensus sequence for the detection of 5′ terminus of RusV genomes originating from Germany, Sweden and Austria, as described previously (*3*). Briefly, the panRusV-2 RT-qPCR was performed with AgPath-ID One-Step RT-PCR reagents (Thermo

Fisher Scientific, Waltham, MA, USA), primers RusV_234+ and RusV_323- (final concentration: 0.8 µM each), probe RusV_256_P (0.4 µM), eGFP-specific primers (0.2 µM each) and probe (0.15 µM) (*2*), and 2.5 µL extracted RNA in a total volume of 12.5 µL. All primers and probes are listed in Appendix Table 2. The reaction was performed with the following cycler setup: 45°C for 10 min, 95°C for 10 min, 45 cycles of 95°C for 15 sec, 60°C for 30 sec and 72°C for 30 sec. A standard RNA preparation of a RusV-positive donkey brain (GenBank accession number MN552442.2) (*4*) served as positive control and was used for the calibration of cycle of quantification (Cq) values in each RT-qPCR analysis.

Sequencing and Phylogenetic Analysis

For phylogenetic analysis, partial p150-encoding sequences of 409 nucleotides (nt) length (corresponding to positions 100 to 508 of reference genome MN552442.2) were generated for all three RusV-positive animals by Sanger sequencing of four overlapping RT-PCR amplicons of 142 to 191 base pairs (bp) length. Briefly, 2.5 µl extracted RNA were amplified in a total volume of 25 µl using the SuperScript III One-Step RT-PCR system with Platinum Taq DNA polymerase (Thermo Fisher Scientific, Waltham, MA, USA) and 0.4 µM each of the respective forward and reverse primers (Appendix Table 2). The cycler setup consisted of 50°C for 30 min, 94°C for 2 min, followed by 40 cycles of 94°C for 30 sec, 63°C for 30 sec, and 68°C for 15 sec, and a final elongation step at 68°C for 5 min. Following separation and visualization by gel electrophoresis, amplification products were purified using Zymoclean Gel DNA Recovery Kit (Zymo Research, Freiburg, Germany) and Sanger sequencing service was provided by Eurofins Genomics (Ebersberg, Germany). Overlapping amplicons were sequenced in both directions and consensus sequences were generated after de novo assembly of quality- and primer-trimmed raw sequences in Geneious Prime 2021.0.1 (Biomatters Ltd, Auckland, New Zealand). Sequences are available in GenBank under accession numbers OQ319478 to OQ319480.

Phylogenetic analysis was performed for the sequences generated in this study in combination with all previously published RusV sequences for which the partial p150-encoding sequences of 409 nt length were available in GenBank (n = 49). Following nucleotide sequence alignment using MUSCLE 3.8.425 (available in Geneious Prime 2021.0.1), a Maximum-likelihood (ML) phylogenetic tree was calculated using IQ-TREE version 2.2.0 (TIM2+F+G4 with 1,000 bootstrap replicates) (*5*).

Immunohistochemistry (IHC)

Appendix Table 3 provides an overview of primary antibodies used in this study, their target structure as well as information about their clonality, host species, performed antigen

retrieval and the source. Immunohistochemistry was performed as previously described (*6*). Briefly, deparaffinization and rehydration was followed by inhibition of endogenous peroxidase activity by incubation in 85% ethanol and 0.5% hydrogen peroxidase for 30 min at room temperature. After washing in phosphate-buffered saline (PBS), heat-induced epitope retrieval was performed by using the respective buffer in a microwave at 800 W for 20 min (Appendix Table 3). For labeling with antibody 9D5, sections were incubated with Proteinase K [1000 ml PBS admixed with 3 μl Proteinase K (20 mg/mL, Roche Diagnostics, Mannheim, Germany)] for 7 min at room temperature. Subsequent washing in PBS was followed by reducing unspecific binding reaction by incubation with inactivated normal goat serum (diluted 1:5 in PBS) for 30 min at room temperature. Primary antibodies were diluted in PBS with addition of 1% bovine serum albumin. Afterwards, sections were incubated with primary antibodies in their respective concentration at 4°C overnight (Appendix Table 3). Negative controls were generated by replacement of the primary antibody with serum of Balb/c mice. After washing in PBS, a biotinylated goat-anti mouse antibody (diluted 1:200 in PBS) was applied and incubated for 60 min at room temperature. Afterwards, the avidin-biotin-peroxidase complex (ABC, Vectastain ABC Kit Standard, Vector Laboratories, Burlingame, USA) was added for 30 min at room temperature. For visualization of immunopositive staining, incubation of ABC was followed by the application of 3,3-diaminobenzidine tetrahydrochloride (0.05%, Sigma Aldrich Chemie GmbH, Darmstadt, Germany) with 0.03% hydrogen peroxide for 5 min at room temperature. Finally, sections were dehydrated and subsequently counterstained with Mayer´s hematoxylin (Roth C. GmbH & Co KG, Karlsruhe, Germany).

In Situ Hybridization (ISH)

ISH was performed by using the ViewRNA ISH Tissue Core Kit (Invitrogen by Thermo Fisher Scientific, Vienna, Austria) and a commercially produced probe (ViewRNA Type 1 probe set, Life Technologies GmbH, Darmstadt, Germany). The probe was designed on the partial p150-encoding RusV sequence available from lions 2 and 3 (GenBank accession numbers OQ319479 and OQ319480). Experimental procedure was performed according to the manufacturer´s protocol with minor variations (*7*). The sections were heated in a dry oven at 60°C for one hour the day before ISH was performed. After deparaffinization and rehydration, sections were incubated in Pretreatment Solution at 85–90°C for 10 or 20 min, depending on the type of tissue. After performing several washing steps in autoclaved double-distilled water and PBS, protease digestion and fixation was achieved by incubation of the sections in protease solution (protease diluted 1:100 in PBS) for 10 min at 40°C.

Afterwards, sections were washed twice in PBS and fixed in 10% NBF for 4 min at room temperature. Using the standard protocol, a positive signal could not be obtained in any animal by ISH. By extending the hybridization time from 2 hours to 4 hours and the application of an additional pretreatment with hydrochloric acid (0.2 M) for 10 min at room temperature, positive reactions were observed. Sections were hybridized with the probe diluted 1:25 in Probe Set Diluent at 38°C. For negative controls, only Probe Set Diluent was applied. Signal detection and amplification was achieved by subsequent incubation with Pre-amplifier Mix, Amplifier Mix and Label Probe 1-AP Solution (diluted 1:500) at 40°C and subsequent application of AP Enhancer Solution for 5 min at room temperature. Finally, sections were stained by incubation with Fast Red Staining Solution for 60 min at 40°C. Counterstaining was performed with Mayer´s hematoxylin (Roth C. GmbH & Co KG).

Appendix Table 1. Detection of rustrela virus in tissue from lions with and without nonsuppurative meningoencephalitis*

					Detection of RusV RNA or antigen			IHC for dsRNA		
No.	Internal ID	Sex	Histopathologic lesions in the CNS	Tested tissue	RT-qPCR Cq value	IHC	ISH	K1	J2	9D5
1	S6659/80	F	Mild, multifocal, lymphohistiocytic ME and vasculitis	Cerebrum, cerebellum	**31, 32**	**Pos**	Neg	**Pos**	**Pos**	Neg
				Liver, spleen	ND	ND	Neg	ND	ND	ND
2	S6076/89	F	Mild, multifocal, lymphohistiocytic ME and vasculitis	Cerebrum, cerebellum	**30, 38**	**Pos**	**Pos**	**Pos**	**Pos**	Neg
				Liver, thyroid gland, lung, pancreas, adrenal gland, heart, stomach, urinary bladder	ND	ND	Neg	ND	ND	ND
3	S6077/89	M	Mild, multifocal, lymphohistiocytic ME and vasculitis	Cerebrum, cerebellum	**29, 29**	**Pos**	**Pos**	**Pos**	**Pos**	Neg
				Kidney, liver, stomach, thymus, urinary bladder, lung, adrenal gland, spleen, heart	ND	ND	Neg	ND	ND	ND
4	S96/06	F	No significant lesions	Cerebrum, cerebellum	Neg	Neg	ND	**Pos**	**Pos**	Neg
5	S692/06	F	No significant lesions	Cerebrum, cerebellum	Neg	Neg	ND	**Pos**	**Pos**	Neg
6	S303/14	M	No significant lesions	Cerebrum, cerebellum	Neg	Neg	ND	**Pos**	**Pos**	Neg
7	S1085/16	F	No significant lesions	Cerebrum, cerebellum	Neg	Neg	ND	**Pos**	**Pos**	**Pos**
				Spleen, adrenal gland, liver, thyroid gland, pituitary gland, kidney, urinary bladder, diaphragm, tongue, pancreas, lung, stomach, intestine, bone marrow, heart, mesenterial lymph node, lung lymph node	ND	ND	Neg	ND	ND	ND
8	S303/19	F	No significant lesions	Cerebrum, cerebellum	Neg	Neg	ND	**Pos**	**Pos**	Neg
9	S885/20	F	No significant lesions	Cerebrum, cerebellum	Neg	Neg	ND	**Pos**	**Pos**	**Pos**
10	S342/21	F	No significant lesions	Cerebrum, cerebellum	Neg	Neg	ND	**Pos**	**Pos**	Neg
11	S32/22	M	No significant lesions	Cerebrum, cerebellum	Neg	Neg	ND	**Pos**	**Pos**	Neg

*Bold text indicates positive result. CNS, central nervous system; Cq, cycle of quantification; dsRNA, double-stranded RNA; F, female; IHC, immunohistochemistry; ISH, in situ hybridization; M, male; ME, meningoencephalitis; ND, not done; Neg, negative; Pos, positive; RT-qPCR, reverse transcription quantitative PCR; RusV, rustrela virus.

Appendix Table 2. Primers and probes used in study of rustrela virus in lions with nonsuppurative meningoencephalitis*

Assay	Primer/probe	Sequence (5′ to 3′)	Reference
panRusV-2	RusV_234+	CCCCGTGTTCCTAGGCAC	Matiasek et al. (*3*)
	RusV_256_P	FAM-GTGAGCGACCACCCAGCACTCCA-BHQ1	Matiasek et al. (*3*)
	RusV_323-	TCGCCCCATTCWACCCAATT	Matiasek et al. (*3*)
eGFP mix 1	EGFP-1-F	GACCACTACCAGCAGAACAC	Hoffmann et al. (*2*)
	EGFP-Probe1_HEX	HEX-AGCACCCAGTCCGCCCTGAGCA-BHQ1	Hoffmann et al. (*2*)
	EGFP-2-R	GAACTCCAGCAGGACCATG	Hoffmann et al. (*2*)
Conventional RT-PCR & Sanger sequencing	RusV_80+	GTCGAGGAGCAGATAAGCCC	Matiasek et al. (*3*)
	RusV_248-	TGCCTARGAACACGGGGCG	This study
	RusV_182+	GARTGCATGAGCGCCGAAGG	This study
	RusV_323-	TCGCCCCATTCWACCCAATT	Matiasek et al. (*3*)
	RusV_227+	CCGCGCCCCGTGTTCYTAGG	This study
	RusV_382-	CCGYTGGGCGAGGCGTADSA	This study
	RusV_338+	TGCCTVGTBAACCCCAGCCC	This study
	RusV_528-	AGCGYCGGGTCYGTVACAAC	This study

*RT-qPCR, reverse transcription quantitative PCR; RusV, rustrela virus.

Appendix Table 3. Overview of primary antibodies used in study of lions with rustrela virus*

Antigen	Target structure	Clonality species	Dilution	Antigen retrieval	Reference
2H11B1	RusV capsid protein	Monoclonal mouse	1:100	Microwave; citrate buffer	Matiasek et al. (*3*)
K1	dsRNA	Monoclonal mouse	1:150	Microwave; citrate buffer	Störk et al. (*6*)
J2	dsRNA	Monoclonal mouse	1:300	Microwave; citrate EDTA buffer	Störk et al. (*6*)
9D5	dsRNA	Monoclonal mouse	1:100	Proteinase K	Störk et al. (*6*)

*dsRNA, double-stranded RNA; EDTA, ethylendiamintetraacetate; RusV, rustrela virus.

References

1. Truyen U, Stockhofe-Zurwieden N, Kaaden OR, Pohlenz J. A case report: encephalitis in lions. Pathological and virological findings. Dtsch Tierarztl Wochenschr. 1990;97:89–91. PubMed

2. Hoffmann B, Depner K, Schirrmeier H, Beer M. A universal heterologous internal control system for duplex real-time RT-PCR assays used in a detection system for pestiviruses. J Virol Methods. 2006;136:200–9. PubMed https://doi.org/10.1016/j.jviromet.2006.05.020

3. Matiasek K, Pfaff F, Weissenböck H, Wylezich C, Kolodziejek J, Tengstrand S, et al. Mystery of fatal "staggering disease" unravelled: novel rustrela virus causes severe meningoencephalomyelitis in domestic cats. Nat Commun. 2023;14:624. PubMed https://doi.org/10.1038/s41467-023-36204-w

4. Bennett AJ, Paskey AC, Ebinger A, Pfaff F, Priemer G, Höper D, et al. Relatives of rubella virus in diverse mammals. Nature. 2020;586:424–8. PubMed https://doi.org/10.1038/s41586-020-2812-9

5. Minh BQ, Schmidt HA, Chernomor O, Schrempf D, Woodhams MD, von Haeseler A, et al. IQ-TREE 2: New Models and Efficient Methods for Phylogenetic Inference in the Genomic Era. Mol Biol Evol. 2020;37:1530–4. PubMed https://doi.org/10.1093/molbev/msaa015

6. Störk T, de le Roi M, Haverkamp AK, Jesse ST, Peters M, Fast C, et al. Analysis of avian Usutu virus infections in Germany from 2011 to 2018 with focus on dsRNA detection to demonstrate viral infections. Sci Rep. 2021;11:24191. PubMed https://doi.org/10.1038/s41598-021-03638-5

7. Postel A, Hansmann F, Baechlein C, Fischer N, Alawi M, Grundhoff A, et al. Presence of atypical porcine pestivirus (APPV) genomes in newborn piglets correlates with congenital tremor. Sci Rep. 2016;6:27735. PubMed https://doi.org/10.1038/srep27735
